# Diphtheria or Streptococcal Pharyngitis: A Case Report Highlighting the Diagnostic Dilemma in the Post-vaccination Era

**DOI:** 10.7759/cureus.6190

**Published:** 2019-11-18

**Authors:** Venkataramana Kandi, Ritu Vaish

**Affiliations:** 1 Clinical Microbiology, Prathima Institute of Medical Sciences, Karimnagar, IND; 2 Microbiology, Prathima Institute of Medical Sciences, Karimnagar, IND

**Keywords:** diphtheria, tonsillitis, vaccine preventable disease, corynebacterium diphtheriae, children

## Abstract

Diphtheria is an acute, highly infectious, toxigenic, and vaccine-preventable disease that commonly affects children under 12 years of age. The incidences of diphtheria have significantly dropped due to vaccination with diphtheria, pertussis, tetani (DPT). Recently, there is an increasing trend in reports of diphtheria throughout the world and specifically from developing countries. According to a World Health Organization (WHO) report, more than 80% of the global diphtheria cases in the post-vaccination era were from India and Indonesia. This could probably be signaling its re-emergence, which may be attributed to several factors that include incomplete immunization. Pharyngitis caused by group A Streptococcus is most frequently seen in children and can be clinically similar in presentation to diphtheria. We share our experience of managing a case of an eight-year-old child, who was clinically suspected to be suffering from diphtheria.

## Introduction

Diphtheria is a bacterial infection caused by *Corynebacterium diphtheriae* (*C*. *diphtheriae*). It is transmitted among humans through the respiratory route (aerosols). Diphtheria is an acute, severely debilitating illness, usually affecting children less than 12 years of age. In diphtheria, there is a formation of thick pseudomembrane or a leathery sheet (diphtheros) on the posterior pharyngeal wall, formed by the accumulation of bacterial cells, epithelial cells, and other inflammatory cells. This causes a mechanical obstruction and results in difficulty in swallowing and, in some cases, dyspnoea (difficulty in breathing). Since *C*. *diphtheriae *is a toxin-producing bacteria, the exo-toxin is released by the bacteria, which then enters the blood/general circulation, resulting in several, other complications in the infected patients. Clinically, diphtheria may present as faucial, laryngeal, cutaneous, and others. *C*. *diphtheriae *has been noted to occur in three different strains/types depending on the intensity of the infection they cause, the gravis type produces a severe infection, the intermedius type results in moderate infection, and the mitis strain causes a mild type of diphtheria [1].

Clinical and laboratory diagnosis assumes great significance to efficiently manage the suspected cases of diphtheria and minimize the resultant morbidity and mortality. Patients with diphtheria usually present with sore throat and fever, which also is the presentation of patients suffering from infection with the more common bacteria, *Streptococcus pyogenes *(beta-hemolytic streptococci/group A streptococci) and other microbial infections [2]. In view of the fact that diphtheria, pertussis, and tetani (DPT) has been in regular use as a vaccine against diphtheria for many years, the clinical cases of diphtheria have almost been negligible. Most pediatricians are now in a dilemma regarding the prevalence of diphtheria and probably misdiagnose the potential cases of diphtheria as a streptococcal sore throat. This type of diagnosis and a delay in the appropriate management of cases of diphtheria may result in severe complications among infected patients and could result in mortality.

Recently, there have been some reports of the re-emergence of diphtheria, which should be considered as a cause of serious concern [3-5]. We report our experience of managing a clinically diagnosed case of diphtheria and emphasize its significance in the era of vaccination.

## Case presentation

An eight-year-old boy was brought to the casualty department attached to the Prathima Institute of Medical Sciences with the chief complaints of fever, malaise, vomiting, and difficulty in swallowing. The boy was admitted to the pediatric intensive care unit (PICU) for further evaluation. The boy’s parents complained of acute onset of low-grade fever three days back. The boy was previously normal and was going to school regularly. The fever episodes were not associated with any type of skin rash. Three to four episodes of vomiting per day were noted along with the fever. The vomiting was non-projectile, non-bilious, blood-tinged, and was stimulated by both solid and liquid food intake. The boy also complained of pain in the throat and had difficulty swallowing. The patient had a loss of appetite, gave a history of high-colored urine, and had generalized weakness.

No previous history of similar complaints in the patient, as well as his two other siblings, was reported. There was no documented evidence/medical record that the patient was immunized with DPT although the parents claimed that the patient was immunized according to the national immunization schedule.

On clinical examination, the patient’s vitals were all good. A noisy breath, probably due to the infection in the throat, was noted, without any dyspnoea. Clinical examination of the pharynx showed grade IV tonsillitis with a grayish-white membranous patch covering the tonsil, which was extending towards the soft palate. The posterior pharyngeal wall revealed congestion, with both sides of the tonsil showing enlargement. The uvula was central, oedematous, showed congestion, and was bleeding on touch.

General physical examination of the patient revealed sunken eyes, loss of the buccal pad of fat, a prominent maxilla, and a scaphoid abdomen. The patient was noted to be underweight (20 kg) as against the recommended weight at the same age (32 kg) and was 133 cm tall as against the recommended height (140 cm) at a corresponding age.

The patient’s parents reported a low-calorie intake of 1200 KCal/day as against the recommended 1920 KCal/day. Also, the patient was only taking 24 g of protein against the daily recommended intake of 38.4 g/day. Considering this, the patient was diagnosed/categorized as suffering from protein-energy malnutrition.

The complete blood picture showed neutrophilic leucocytosis, and borderline platelet counts (1.5 lakh cells/mm^3^). The patient had raised C-reactive protein (CRP) (2.4 mg/dL) and erythrocyte sedimentation rate (ESR) (40 mm).

**Figure 1:**
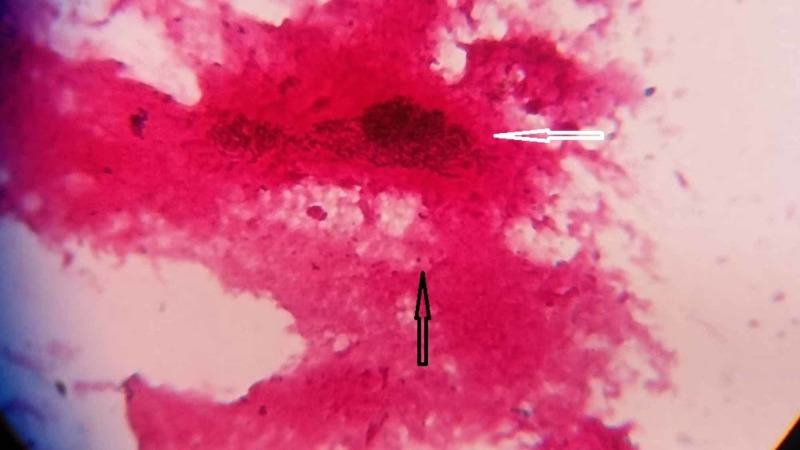
Direct Gram’s stain of the throat swab showing plenty of Gram-positive bacilli (white arrow) with occasional Gram-positive cocci (black arrow)

A preliminary diagnosis of grade IV tonsillitis was made, and a throat swab was sent to the clinical microbiology laboratory for a direct Gram's stain, culture, and sensitivity. On direct Gram’s stain of the throat swab, plenty of Gram-positive bacilli were observed, with occasional Gram-positive cocci, as shown in Figure 1.

**Figure 2:**
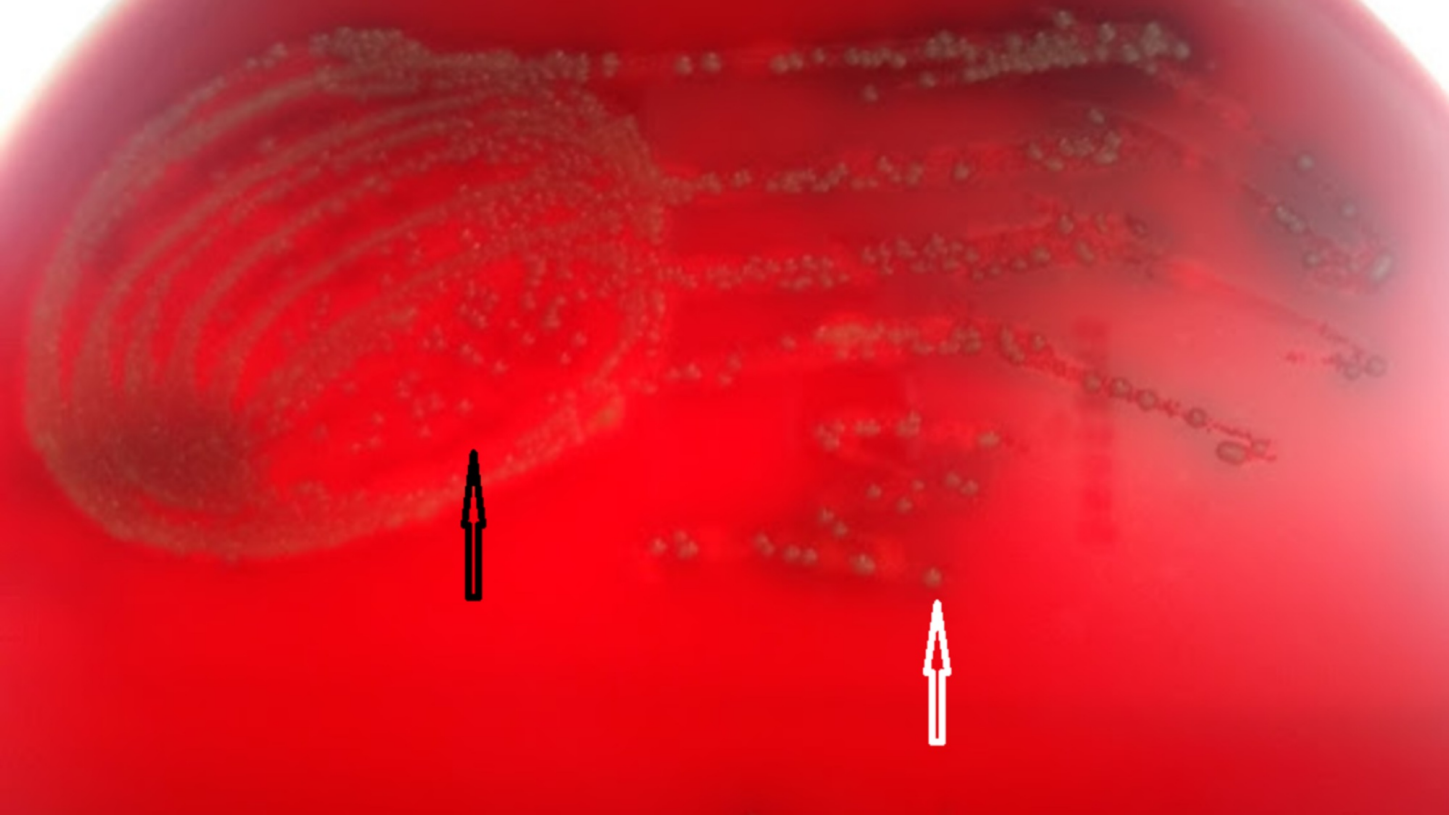
Culture on blood agar showing opaque, non-hemolytic colonies (white arrow) and translucent beta-hemolytic colonies (black arrow)

Culture on blood agar showed 2-3 millimeter, small, round, white-cream-colored non-hemolytic colonies along with 1-2-millimeter, pinpoint, translucent beta-hemolytic colonies, as shown in Figure 2.

**Figure 3:**
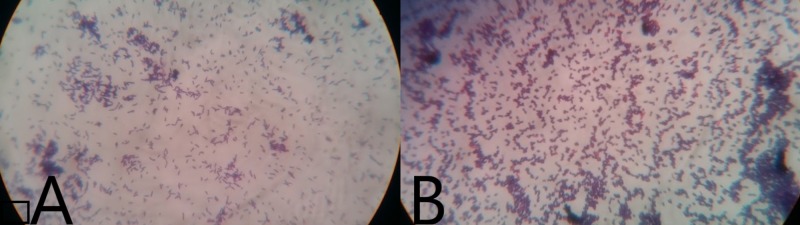
Gram’s stain of the small colonies showing gram-positive bacilli (A) and the pinpoint colonies showing gram-positive cocci (B)

Gram’s stain of the small and the pinpoint colonies revealed gram-positive bacilli and gram-positive cocci in pairs respectively as shown in Figure 3.

**Figure 4:**
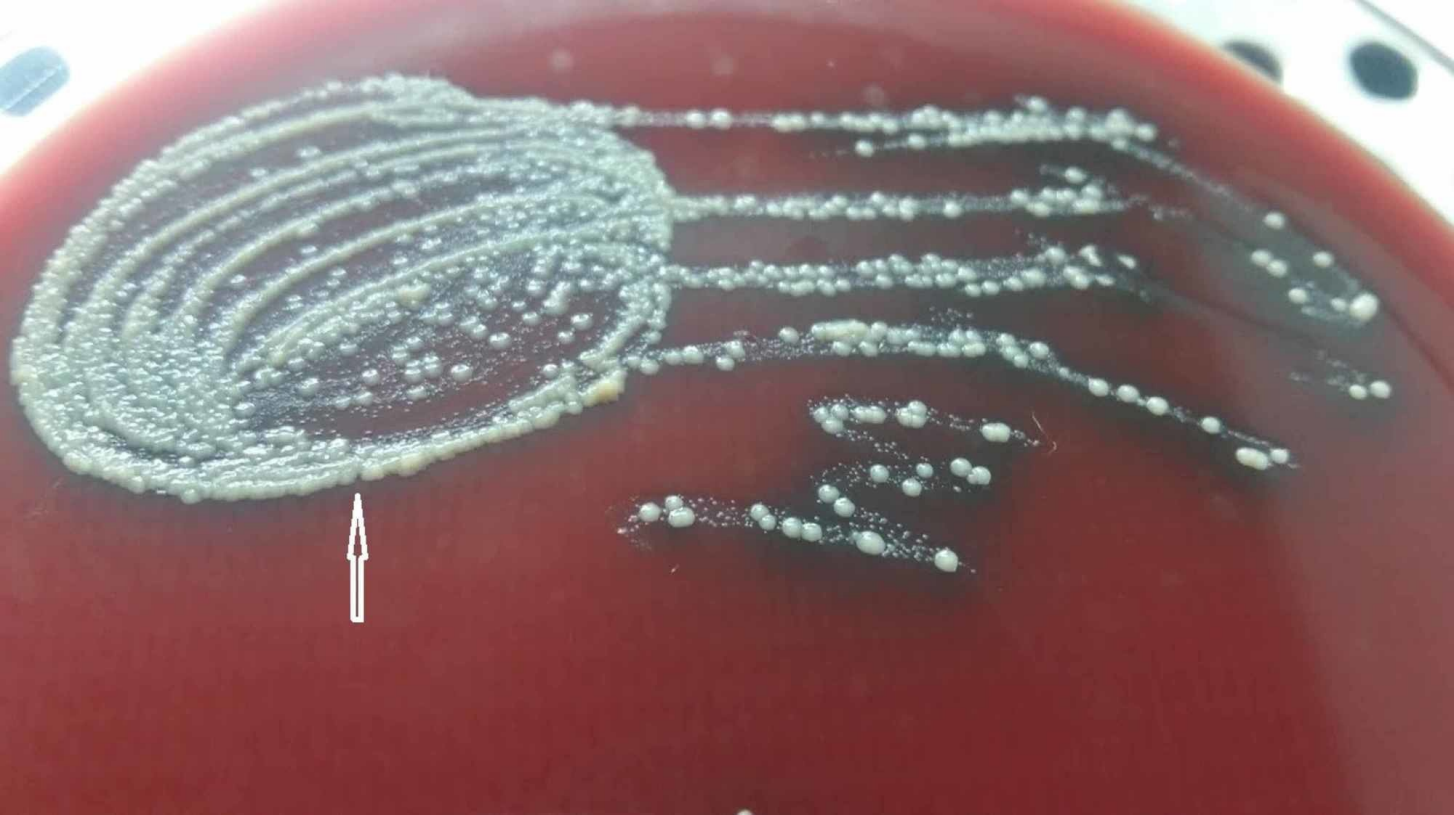
White to cream-colored, raised, glossy, and butyrous colonies (white arrow) of diphtheria like bacteria on blood agar

The colonies on blood agar were non-hemolytic (*C*. *diphtheriae *forms hemolytic colonies), glossy (gravis type is matt-like) in appearance, raised (intermedius are flat), and were glistening and butyrous (butter-like) in texture, as shown in Figure 4.

They were catalase-positive, non-motile, and non-fermenters on triple sugar iron agar (TSI). The Gram-positive bacilli were identified as morphologically and biochemically resembling *C*. *diphtheriae*. The gram-positive cocci were catalase-negative and were identified as *Streptococcus *species (*Streptococcus *Spp).

**Figure 5:**
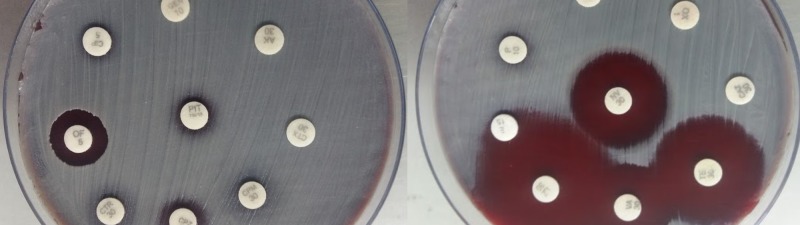
Antimicrobial sensitivity pattern of diphtheria-like bacteria by the Kirby-Bauer disk diffusion method

Both the gram-positive cocci and the bacilli showed varied sensitivity patterns using the Kirby-Bauer disk diffusion method. The antimicrobial susceptibility pattern of gram-positive bacilli showed sensitivity to vancomycin, linezolid, tetracycline, and ofloxacin. Resistance was observed against penicillin, oxacillin, clindamycin, ciprofloxacin, cefotaxime, cefepime, cefoperazone, ceftriaxone, ceftazidime, amikacin, gentamicin, and piperacillin-tazobactam, as shown in Figure 5.

**Figure 6:**
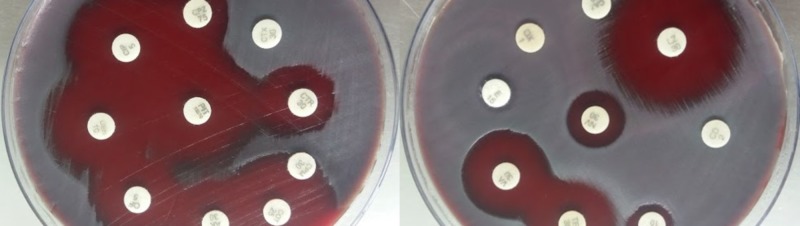
Antimicrobial sensitivity pattern of Streptococcus species by the Kirby-Bauer disk diffusion method

The *Streptococcus *spp. isolated was sensitive to amikacin, gentamicin, ciprofloxacin, ofloxacin, trimethoprim-sulfamethoxazole, vancomycin, linezolid, tetracycline, and piperacillin-tazobactam. Resistance was noted against penicillin, erythromycin, clindamycin, oxacillin, cefotaxime, cefepime, cefoperazone, ceftriaxone, and ceftazidime, as shown in Figure 6.

Considering the transmissibility of the infection, the patient was put under isolation. Treatment was initiated with 80,000 units of diphtheria antitoxin through the intravenous route. Since the isolated organisms were resistant both to penicillin and erythromycin, the drugs of choice in the case of suspected diphtheria, and because the patient also had beta-hemolytic Streptococci, the patient was started on a course of piperacillin-tazobactam and amikacin. The patient had a gradual and uneventful recovery.

The bacterium morphologically resembling *C*. *diphtheriae *was not confirmed using the standard anti-toxin by the Elek’s gel precipitation test due to the unavailability of a suitable identification system. Also, the close household contacts were neither screened nor administered prophylactic antibiotics as suggested/recommended by the World Health Organization (WHO) because of the non-cooperation of the patient's parents.

## Discussion

Diphtheria is a highly infectious and reportable bacterial disease that is prevalent throughout the world. The introduction and success of the DPT vaccination had been instrumental in the control of the disease, which mostly affects children below 12 years of age, causing significant morbidity and mortality. Streptococcal sore throat, caused by group A Streptococci is another upper respiratory tract infection prevalent among children. The clinical presentation of both diphtheria and streptococcal pharyngitis appears similar, and the clinical diagnosis becomes difficult. Assuming that the cases of diphtheria are almost negligible due to DPT vaccine, and with limited knowledge of the prevalence of diphtheria in the post-vaccination era, most physicians/pediatricians may misdiagnose the diphtheria cases as streptococcal infections. Such diagnoses may result in the spread of diphtheria among the contacts and also delay the initiation of treatment.

Global scenario of diphtheria

The re-emergence of diphtheria has been a point of discussion almost since the past decade. The occurrence of diphtheria in the post-vaccination era was attributed to the discrepancies (incomplete vaccination) in the immunization. Most infections in the post-vaccination era have been noted to emerge from the developing nations, which include the incidences of outbreaks from Indonesia, Bangladesh, and Yemen [6-9].

Isolated reports of outbreaks of diphtheria have also been reported from the developed nations, including the United States of America (USA). Even such reports of outbreaks were attributed to low socioeconomic conditions similar to those observed in the developing nations [10-12].

Indian scenario of diphtheria

Recent reports of outbreaks and isolated case reports from developing countries like India reassert the fact that there is a possibility of the re-emergence of diphtheria in the post-vaccination era [13-15]. Also, reports of incidences of diphtheria from the combined state of Andhra Pradesh and the separated state of Telangana (India) support the fact that the infection is prevalent and that the pediatricians need to be cautious while diagnosing the suspected patients [16-17].

The epidemiological data of diphtheria in India appears to be inadequate. A recent article reported an analysis of diphtheria in India over the past two decades (1996-2016) [18]. This report suggested that diphtheria cases are frequent among school-going children and adolescents in India. The study had also noted that there was an 80% coverage of the initial three doses of vaccine and that there is no reliable data on the coverage of the booster dose. This report also observed that India accounts for more than half of the diphtheria cases reported worldwide (2001-2015). It also confirms that most states in India had reported outbreaks/cases of diphtheria, and amongst all, the combined Andhra Pradesh and Telangana reported an increased frequency of diphtheria cases (>1000 cases/year between 2005 and 2014) [18].

**Figure 7:**
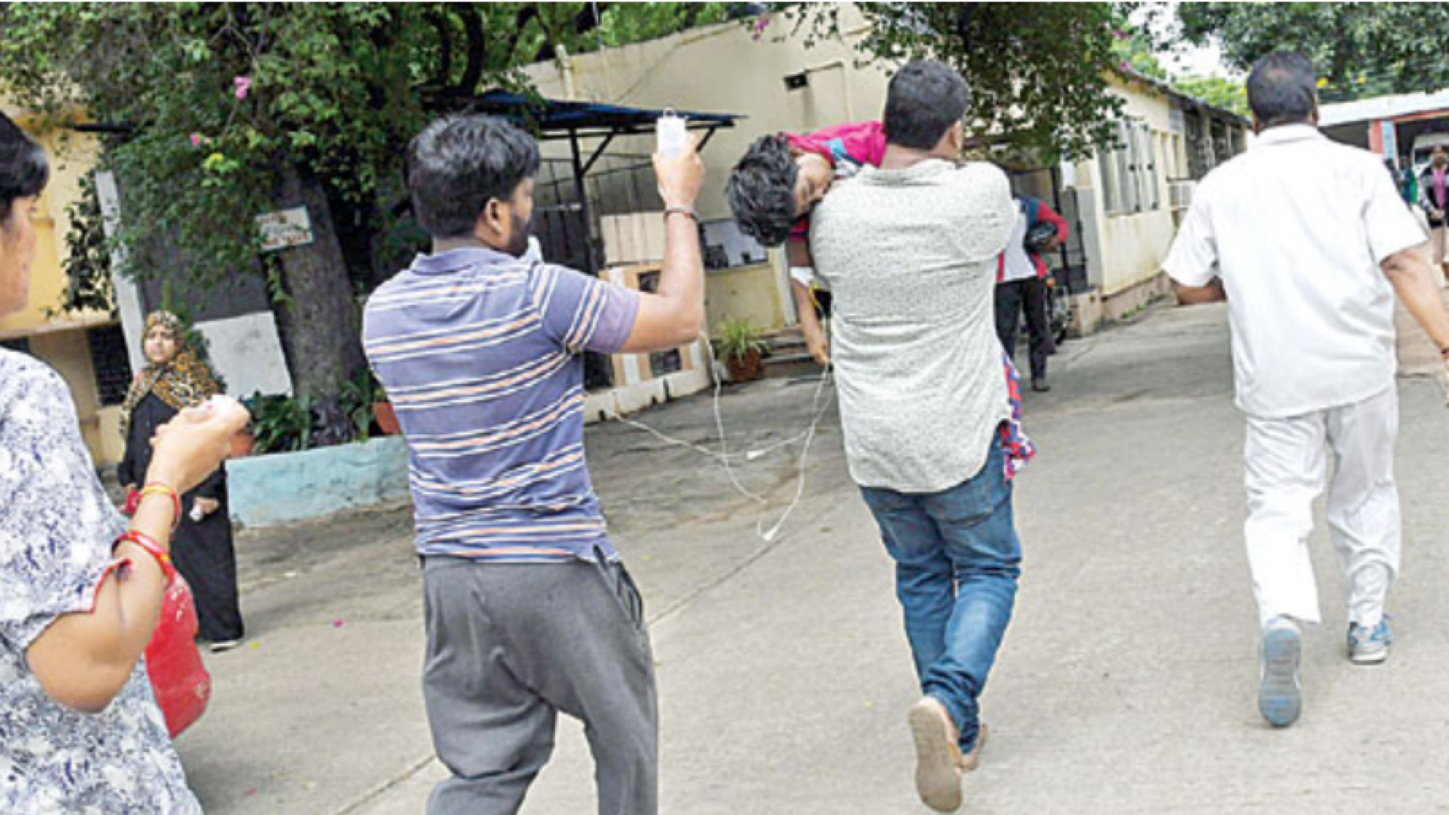
A child possibly suffering from diphtheria is being carried to a better medical facility by the relatives

There have been several recent reports of outbreaks and newspaper articles highlighting the seriousness of the present situation, which emphasizes the role of the public, healthcare workers, and governments in order to control and prevent the spread of diphtheria [19-20]. A local newspaper published (in the Telegu language) a picture of parents and relatives carrying and transporting a pediatric patient to a better medical facility whose condition worsened with suspected diphtheria, as shown in Figure 7.

Diphtheria has been controlled to a great extent with the introduction of DPT throughout the world. In spite of the vaccination, several studies in the past have reported the incidences of diphtheria globally. The condition in developing and financially constrained third-world countries appears to be worse due to illiteracy, malnutrition, overcrowding, and inadequate immunization. Isolated clinical cases and frequent reports of outbreaks of diphtheria-like infections should be adequately addressed in order to eliminate the infection. The governments should, therefore, actively perform surveillance of immunization as well as document the burden of morbidity and mortality associated with diphtheria.

## Conclusions

The eight-year-old boy who presented with symptoms of fever, sore throat, and thickening of the posterior pharyngeal wall was provisionally diagnosed as a possible case of diphtheria. The laboratory confirmation of diphtheria was not possible because of inadequate facilities. The diagnosis was based on careful clinical and laboratory observations. The patient was isolated from others to avoid contact infections and was successfully treated with antibiotics and antidiphtheritic serum.
